# When Language Switching has No Apparent Cost: Lexical Access in Sentence Context

**DOI:** 10.3389/fpsyg.2013.00278

**Published:** 2013-05-30

**Authors:** Jason W. Gullifer, Judith F. Kroll, Paola E. Dussias

**Affiliations:** ^1^Department of Psychology, Pennsylvania State University, University ParkPA, USA; ^2^Center for Language Science, Pennsylvania State University, University ParkPA, USA; ^3^Department of Spanish, Italian, and Portuguese, Pennsylvania State University, University ParkPA, USA

**Keywords:** bilingualism, language switching, switch costs, lexical access, sentence context, cognates

## Abstract

We report two experiments that investigate the effects of sentence context on bilingual lexical access in Spanish and English. Highly proficient Spanish-English bilinguals read sentences in Spanish and English that included a marked word to be named. The word was either a cognate with similar orthography and/or phonology in the two languages, or a matched non-cognate control. Sentences appeared in one language alone (i.e., Spanish or English) and target words were not predictable on the basis of the preceding semantic context. In Experiment 1, we mixed the language of the sentence within a block such that sentences appeared in an alternating run in Spanish or in English. These conditions partly resemble normally occurring inter-sentential code-switching. In these mixed-language sequences, cognates were named faster than non-cognates in both languages. There were no effects of switching the language of the sentence. In Experiment 2, with Spanish-English bilinguals matched closely to those who participated in the first experiment, we blocked the language of the sentences to encourage language-specific processes. The results were virtually identical to those of the mixed-language experiment. In both cases, target cognates were named faster than non-cognates, and the magnitude of the effect did not change according to the broader context. Taken together, the results support the predictions of the Bilingual Interactive Activation + Model (Dijkstra and van Heuven, 2002) in demonstrating that bilingual lexical access is language non-selective even under conditions in which language-specific cues should enable selective processing. They also demonstrate that, in contrast to lexical switching from one language to the other, inter-sentential code-switching of the sort in which bilinguals frequently engage, imposes no significant costs to lexical processing.

## Introduction

Bilinguals activate words in both of their languages even when they consciously intend to use one language alone (e.g., Duyck et al., 2007; see Dijkstra, 2005, for a review). Despite the parallel activation of both languages, lexical switching, from one language to the other, incurs processing costs that have been the subject of debate in the literature on bilingual language processing (e.g., Green, 1998; Meuter and Allport, 1999; Finkbeiner et al., 2006). In contrast, when lexical switching is under the control of the speaker, switch costs are reduced or absent (e.g., Gollan and Ferreira, 2009). In discourse, proficient bilinguals can also code-switch with one another, moving in and out of each of their two languages even in the middle of a sentence, with relatively few overt processing costs (e.g., Poplack, 1980). The fluency with which bilinguals use each of their languages regardless of whether one or both languages are required, suggests that they have a highly developed mechanism of cognitive control (e.g., Abutalebi and Green, 2007; Abutalebi et al., 2012).

The parallel activation of the two languages is a pervasive phenomenon and a hallmark of the interactivity within the bilingual lexicon. It is observed most reliably during the processing of words that share form in their two languages (e.g., cognates: the word “piano” in English and Spanish or interlingual homographs: “pie” in Spanish means *foot*, not the baked-good). Bilinguals, but not monolinguals, show differential processing patterns within a single-language for these form-overlapping words compared to lexically matched non-overlapping words, suggesting that the lexical representations of both languages are activated for the use of a single-language alone (e.g., Dijkstra, 2005). The absence of such effects in monolingual speakers indicates that the differential processing is due to bilingualism and not to lexical variation.

Recent work has investigated whether there are linguistic cues that can allow bilinguals to selectively access words in a single-language without influence from the unintended language. Such cues might theoretically include linguistic information that is specific to one language. The presence of linguistic cues alone is not sufficient to curb activation from the unintended language. For bilinguals, the activation of the language not in use is evident despite the presence of language-specific orthography (e.g., Van Assche et al., 2009), cues related to the accentedness of speech (e.g., Lagrou et al., 2011), and informative sentence contexts (e.g., Schwartz and Kroll, 2006; Duyck et al., 2007; Libben and Titone, 2009). These cues to the language in use appear to be insufficient to bias processing to that language alone. The only factor that has been shown to reduce the activation of the language not in use is a strongly biased semantic context. When sentences are highly semantically constrained such that upcoming words are highly predictable, the processing of language-ambiguous words such as cognate, or homographs becomes more similar to control words (e.g., Schwartz and Kroll, 2006; Van Hell and de Groot, 2008; Libben and Titone, 2009; but see Van Assche et al., 2010). At issue is whether these language-specific constraints operate early in processing to bias selection of the intended language or late in processing once the two languages have become activated.

The Bilingual Interactive Activation Plus (BIA+) model of word recognition (Dijkstra and van Heuven, 2002) proposes a late account of language selection by assuming that words in the two languages are stored in an integrated lexicon and that task demands (e.g., language of the task) do not influence the earliest stages of word recognition. On this account, language cues function to distinguish different language alternatives only after activation of both languages has occurred. Eye-tracking evidence provides support for this late account of language selection by showing that semantic constraints decrease cognate effects in late but not early measures of fixation duration (e.g., Libben and Titone, 2009).

If the parallel activation of the bilingual’s two languages is only resolved relatively late in lexical processing, then we might predict language switching would be relatively cost-free. If both languages are active regardless of the requirements of the task, then forcing the activation of the language not in use by mixing two languages or switching from one language to the other should have little consequence. Despite the evidence for language non-selectivity, studies of both bilingual word recognition and production show that there are processing costs when bilinguals switch languages. Switch costs are quite robust in comprehension (e.g., Grainger and Beauvillain, 1987; Thomas and Allport, 2000; Von Studnitz and Green, 2002) and also in production (MacNamara et al., 1968; Meuter and Allport, 1999; Costa and Santesteban, 2004; Costa et al., 2006; Gollan and Ferreira, 2009). The presence of switch costs suggests that the appropriate language for a given trial is activated and influences processing despite its apparent inability to function as a cue during word recognition.

In word recognition, switch costs tend to be of the same magnitude regardless of whether switching into the L1 or the L2 (e.g., Thomas and Allport, 2000; Von Studnitz and Green, 2002). Asymmetries in switch costs during word recognition have been revealed using time-course sensitive measures such as event-related potentials (e.g., Jackson et al., 2004). In word production, the magnitude of switch costs are frequently asymmetric with a larger cost observed for switching into the L1 after speaking the L2 compared to the reverse case (e.g., Meuter and Allport, 1999). Asymmetric language switch costs in production have been taken to reflect the role of inhibitory control during language selection (e.g., Green, 1998). The more dominant L1 may require strong inhibition during the production of the weaker L2. Comparatively, the weaker L2 is less strongly activated during L1 use, and thus requires less inhibition. Hence, the amount of inhibition that must be overcome for a switch into the L2 is less compared to a switch into the L1 (but see Costa et al., 2006).

Lexical switch costs in production are present regardless of whether the switch is imposed upon the bilingual (e.g., Meuter and Allport, 1999) or under the control of the bilingual (e.g., Gollan and Ferreira, 2009). The magnitude of switch costs may vary under these conditions, but costs are present regardless of the predictability of switches (e.g., MacNamara et al., 1968) and they are present regardless of the preparation time between the language cue and the stimulus to be named (Costa and Santesteban, 2004). However, tasks involving voluntary switching or an increased preparation time can result in a decreased magnitude of the switch cost (e.g., MacNamara et al., 1968; Costa and Santesteban, 2004). These results suggest that the magnitude of switch costs can be modulated but that switch costs themselves cannot be eliminated.

In contrast to the forced language switching that is typical in laboratory studies at the lexical level, research on bilingual code-switching examines the way that bilingual speakers produce and comprehend language switches that occur in sentence context. Code-switching can occur between sentences, inter-sententially, as well as within sentences, intra-sententially. The choice of when and where to switch languages is governed by sociolinguistic factors (for a review see Quinto-Pozos, 2009) and by grammatical rules or patterns (Poplack, 1980; Lipski, 1985; Di Sciullo et al., 1986; Myers-Scotton, 1993). Critically, naturalistic and laboratory studies of code-switching provide support for the claim that switch costs may not always be costly. Corpus studies show that there are many communities of bilinguals who code-switch frequently between two languages (e.g., Pfaff, 1979; Poplack, 1980; Lipski, 1985). A study of pauses during one bilingual’s free speech suggests that code-switched utterances exhibit pauses in comparable quantity and duration as compared to unilingual speech (Timm, 1983). Not all groups of bilinguals choose to code-switch within sentences, but all bilinguals have experience switching languages between sentences, making inter-sentential switching the most common form of code-switching.

The present study bridges the gap between experimental and linguistic studies on language switching by asking how switching languages inter-sententially in a laboratory setting modulates the ease of recognizing words embedded within those sentences. Crucially for our purposes, most experimental studies on language switching have focused on switching between individual words in each language outside of sentence context (i.e., Meuter and Allport, 1999; Costa et al., 2006; Gollan and Ferreira, 2009) and although some psycholinguistic studies have examined language switches at the grammatical level (e.g., Hatzidaki et al., 2011) and at the lexical level in a mixed-language environment (e.g., intra-sentential: Altarriba et al., 1996; Moreno et al., 2002; Proverbio et al., 2004; inter-sentential: Ibáñez et al., 2010; Titone et al., 2011), most do not explicitly examine inter-sentential switch costs.

In the experiments reported here, Spanish-English bilinguals read sentences word-by-word and named a target word embedded in the middle of each sentence while the latency to begin naming was recorded. Word naming latencies were chosen as the dependent measure because word naming ensures that participants have selected a specific word form in the intended language. The critical targets were either cross-language cognates, with similar form and meaning in both languages, or unambiguous control words. In Experiment 1, the language of the sentence changed every two sentences in an alternating runs sequence, creating a mixed-language block. In Experiment 2, the language of the sentence was constant within a given block, so that the language of the task switched only between blocks. If inter-sentential language switching imposes processing costs in a manner similar to the robust findings of lexical switching costs, then the time to name target words embedded in sentence contexts should be slower in switched than non-switched sentences. Naming times should also be slower overall in the mixed-language conditions of Experiment 1 than in the blocked language conditions of Experiment 2. If switching the language of a sentence affects the degree of cross-language activation, then the magnitude of cognate facilitation for target words should also be greater in switched and mixed conditions than in blocked conditions. In contrast, the BIA+ model makes the counterintuitive prediction that there should be no switch costs for words embedded within a sentence because the broader situational context (e.g., language of an experimental task) is hypothesized not to influence early stages of lexical access, and in the current study there is no lexical switch at the point in the sentence at which the target word is named. Likewise, the model predicts that the magnitude of cognate facilitation reflects bottom-up processes that are unaffected by the context in which word recognition occurs. It should be noted that in the mixed-language block, language switches do not occur directly at the target word. Instead, language switching occurs only at the beginning of the sentence. In this regard, the global effects of switching the language of the sentence context are measured as opposed to more local effects of lexical switching. To anticipate the findings, the results indicate that inter-sentential language switching incurs no observable cost to lexical processing when words are embedded in a sentence context, in contrast to studies on lexical switching. The results support the predictions of the BIA+ model of word recognition (BIA+; Dijkstra and van Heuven, 2002) in that the degree of co-activation of the two languages does not appear to depend on the global language context.

## Experiment 1: Mixing the Language of Sentence Context

The purpose of Experiment 1 was to investigate the relationship between inter-sentential language switching and lexical access. Sentences were presented using RSVP (rapid serial visual presentation), and during the presentation of each sentence, bilinguals named designated target words. The critical targets were either cognates or unambiguous control words. In a previous norming study, when the same critical targets were presented to English-Spanish bilinguals for naming out of sentential context, the cognates produced reliable facilitation relative to the controls (Gullifer et al., 2010). In the present experiment, these words were embedded in sentence context and the language of the sentence changed every two sentences (i.e., alternating runs) to form a mixed-language block. If this form of language switching imposes additional processing costs above and beyond those of parallel activation of two languages, then target words embedded in switched sentences might be expected to take longer to name than target words embedded in non-switched sentences. If switching the language of a sentence repeatedly within a block affects the degree of cross-language activation, then the magnitude of cognate facilitation for target words should be greater in switched conditions than in non-switched conditions. In contrast, BIA+ predicts that the task context itself has no influence on the earliest stages of lexical access, so little effect of inter-sentential switching would be predicted. Likewise the magnitude of the cognate effect should not depend on whether the trial is a switch trial or a non-switch trial.

### Methods

#### Participants

Twenty-seven Spanish-English bilinguals participated in the mixed-language experiment. The participants were recruited from the Pennsylvania State University and the State College area, and they were paid $10 per hour for their participation in the experiment. All subjects gave informed consent and the procedures had the approval of the Institutional Review Board of the Pennsylvania State University. All of the bilinguals were native Spanish speakers, having acquired Spanish at birth. Acquisition of English as a second language varied across participants. Fourteen of the participants reported acquiring and using English as a second home language (reported age of acquisition in years: *M* = 6.9) and 13 reported using only Spanish at home (reported age of acquisition in years: *M* = 9.0). Participants completed language history questionnaires to assess subjective language proficiency. Additionally, a picture naming task and portions of English and Spanish grammar tests (Michigan English Language Institute College English Test; English Language Institute, 2001 and the Diploma de Español como lengua extranjera; Ministry of Education, Culture, and Sport of Spain, 2006) were administered to assess objective language proficiency. Finally, an Operation-Span task (i.e., O-Span; Turner and Engle, 1989) and a Simon task (Simon and Rudell, 1967) were administered to assess working memory and cognitive control. The picture naming task included pictures with cognate and non-cognate names and was also used to measure independent evidence for language co-activation in our sample. Participants named the pictures in English. Comprehension question accuracy from the main study was also used to assess relative language proficiency. The full set of participant characteristics from Experiment 1 is shown in Table 1. Some participants have data missing for certain side-tasks (e.g., due to equipment malfunction during the task; see Table 1 for valid *N*). In order to maintain high statistical power, all participants were maintained in the main analysis.

**Table 1:** **Participant characteristics**.

Performance measure	Experiment 1: mixed group			Experiment 2: blocked group		
	Valid *N*	*M*	SD	Valid *N*	*M*	SD
English comprehension performance (%)	27	0.84	0.07	26	0.85	0.11
Spanish comprehension performance (%)	27	0.83	0.09	26	0.83	0.11
Age (in years)	26	26.04	7.90	26	21.92	4.51
Self-ratings: English (out of 10)	26	8.82	0.78	26	8.83	0.92
Self-ratings: Spanish (out of 10)	26	9.34	0.73	26	9.18	0.98
Spanish grammar – DELE score (out of 50)	24	40.42	7.14	24	40.21	6.96
English grammar – MELECIT score (out of 50)	24	37.54	9.94	24	37.42	9.11
English picture naming: average Cognate Latency	21	1029	181	25	1011	162
English picture naming: average Non-cognate Latency	21	1100	174	25	1101	206
English picture naming: average latency	21	1064	170	25	1056	178
English picture naming: cognate accuracy	21	0.93	0.04	26	0.93	0.09
English picture naming: non-cognate accuracy	21	0.87	0.10	26	0.88	0.15
English picture naming: average accuracy	21	0.90	0.06	26	0.90	0.12
Operation-span score (out of 60)	24	34.67	9.43	26	40.35	11.52
Simon score (in ms)	24	45.88	33.17	26	47.14	19.80

*Participant characteristics for Experiments 1 and 2. Performance on comprehension questions in the main task, age, objective and subjective language proficiency measures, and cognitive measures are shown*.

There were no differences in performance on the grammar tests which measured objective language proficiency [English: *M* = 40.42; Spanish: *M* = 37.54; *t*(23) = 1.39, *p* > 0.05, *r*^2^ = 0.08]^1^, and there was no significant difference between English and Spanish in accuracy on the comprehension questions of the main task [English: *M* = 0.84; Spanish: *M* = 0.83; *t*(26) = 0.671, *p* > 0.05, *r*^2^ = 0.02]. Participants produced significant cognate facilitation in RTs in the English picture naming task [cognates: *M* = 1029 ms; non-cognates: *M* = 1100 ms; *t*(20) = 3.253, *p* < 0.05, *r*^2^ = 0.35]. Overall, participants were relatively balanced in the use of their two languages even though the circumstances regarding language acquisition varied (i.e., they were not all early and balanced lifelong bilinguals). Despite the relative balance of the two languages on objective measures of language knowledge and processing, participants tended to rate themselves as being more proficient in Spanish than in English [English: *M* = 8.82; Spanish: *M* = 9.34; *t*(25) = 2.07, *p* < 0.05, *r*^2^ = 0.15].

### Materials

#### Critical words

The critical items consisted of 128 Spanish words and their English translations (see Supplementary Material). Sixty-four words were cognate words between English and Spanish (e.g., *cable*) and 64 were lexically matched non-cognate control words (e.g., *chispa* in Spanish meaning *spark* in English). The cognates and non-cognates were matched on word length, lexical frequency, number of phonemes, and number of syllables within each language. The degree of form overlap of the cognates varied^2^, but the cognates were more orthographically similar (*M* = 0.73) across the two languages relative to the matched control words [*M* = 0.16; *t*(114.60) = 20.07, *p* < 0.001, *r*^2^ = 0.78]. To ensure that any potential cognate facilitation effect observed during the experiment could not be attributed to lexical properties of the stimuli, a monolingual control group named the target words in isolation. For the monolinguals, cognates were named significantly slower (*M* = 521 ms) compared to non-cognate controls [*M* = 513 ms; *t*(16) = 3.3, *p* < 0.001, *r*^2^ = 0.4]. This effect reflected a slight difference in orthographic length of the cognates (*M* = 7.0) and control words [*M* = 6.3; *t*(63) = 2.1, *p* < 0.05, *r*^2^ = 0.07]. After RTs were statistically controlled for orthographic length, the effect of cognate status was no longer significant [*t*(16) = 0.27, *p* > 0.05, *r*^2^ < 0.01]. Because the monolingual “cognate effect” was in the reverse direction compared to what is predicted for bilingual speakers and because the effect appears to be mediated by an effect of orthographic length, it is safe to assume that any facilitatory cognate effect observed for bilinguals reflects parallel activation of the two languages and not a spurious lexical effect. Given the small inhibitory effect for the monolinguals, an observed effect of cognate facilitation for bilinguals would therefore be likely to be an underestimation of the actual effect of language co-activation.

#### Sentences

Sentences were written for each of the 128 target words (see Supplementary Material). The sentences were written with the intention to keep semantic constraint low to avoid introducing potentially confounding effects due to a highly probably target word. Total sentence length and number of words before the critical item were controlled for comparisons between cognates and non-cognates within each language. The mean sentence length did not differ according to word type condition in English [cognates: 17.5; non-cognates: 17.6; *t*(254) = 0.5, *p* > 0.05, *r*^2^ < 0.01] nor did it depend on word type condition in Spanish [cognates: 17.2; non-cognates: 17.1; *t*(254) = 0.4, *p* > 0.05, *r*^2^ = 0.01]. Pre-critical sentence length did not differ between word type condition within English [cognates: *M* = 11.5; non-cognates: *M* = 11.7; *t*(254) = 0.73, *p* > 0.05, *r*^2^ < 0.01] nor did it differ between condition within Spanish [cognates: 11.3; non-cognates: 11.4; *t*(254) = 0.62, *p* > 0.05, *r*^2^ < 0.01]. The final set of experimental sentences consisted of 256 sentences in Spanish and their 256 translations into English (128 English sentences – 64 cognates, 64 controls; 128 Spanish Sentences – 64 cognates, 64 controls). The total set of 512 sentences was then divided into two lists. The sentences were counterbalanced such that the translations of any given sentence did not appear within the same list. However, each list contained a single repetition of each critical word across languages. To distract attention from the goal of the main task, 96 filler sentences were added to each list (48 in each language). Each filler sentence contained a target word to be named and a comprehension question. Care was taken to ensure that none of the words in the filler sentences overlapped with critical target words of the experimental sentences. A control study with monolingual English speakers reading the sentence materials confirmed that there were no effects of cognate status for monolingual speakers [cognates: *M* = 532 ms; non-cognates: *M* = 532 ms; *t*(22) = 0.06, *p* > 0.05, *r*^2^ < 0.01].

English and Spanish sentences were interleaved in alternating runs such that the language of the sentence changed every two sentences (e.g., two English sentences followed two Spanish sentences or vice versa). Interleaving was accomplished via a stochastic computer algorithm that sought to keep the number of occurrences of each condition and the number of transitions between each condition consistent within runs (i.e., within the non-switch trials) and across runs (i.e., within the switch trials), as well as equivalent within and across languages. Six versions of the materials were constructed. The order of sentence language was counterbalanced across participants.

#### Procedure

**Figure 1:**
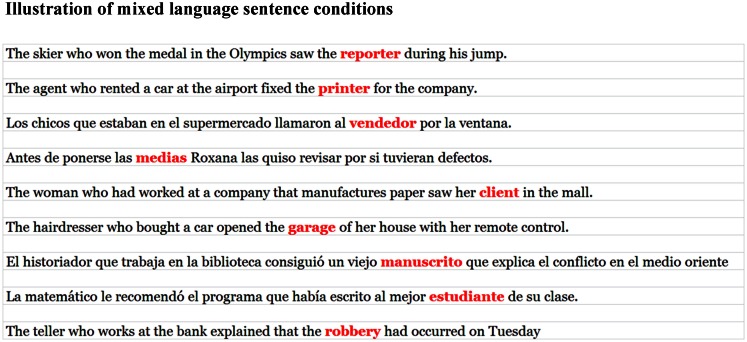
**Sentences were presented one word at a time using Rapid Serial Visual Presentation**. The duration of each non-target word was 300 ms. Target words were marked in red (targets are also bold here) and remained on the screen until they were spoken by the participant.

The experiment lasted for two 1 h long experimental sessions that were carried out over 2 days. At the beginning of each session, participants gave informed consent. During the first session, they completed a language history questionnaire to gauge their language background (including subjective measures of proficiency). Participants were then seated at a computer where they began the sentence task. Sentences were presented using RSVP such that participants read sentences silently word-by-word until they encountered a target word, which was displayed in red. They were instructed to name the target word aloud quickly and accurately. An example of the progression of the experiment is illustrated in Figure 1. Following the main task, the participants were invited back for a follow-up study during which they completed the battery of cognitive and linguistic tasks.

Stimuli were presented on an LCD monitor connected to a computer running Windows XP or Windows 7 and E-Prime 2.0 presentation software (Psychological Software Tools, Inc., Pittsburgh, PA, USA; http://www.pstnet.com). Instructions were displayed and given verbally in English. Following instruction, participants completed 12 practice trials to ensure that they understood the task. Each trial of the RSVP task proceeded as follows. Participants saw a fixation cross that remained on the screen until a button was pressed. Following fixation, sentences were presented one word at a time at a rate of 300 ms per word. The target word of the sentence was marked in red and remained on the screen until the participant spoke the name of the word aloud. A voice trigger was collected using the E-Prime button box and microphone. The trigger advanced the experiment and measured the reaction time (RT) for the onset of articulation. Filler sentences contained yes/no comprehension questions that were answered using the keyboard. Participants were given a break at the half-way point of the experiment.

### Results

Accuracy on the word naming was coded by undergraduate research assistants who spoke English and Spanish. Any trials in which an incorrect word was named or in which the production would add variability to RTs (e.g., hesitation before naming the target word) were removed from the RT analysis. RT data were cleaned using a procedure for the removal of absolute and relative outliers. First, considering only correctly named trials, RTs above 2000 ms and below 200 ms were removed. Next, on the resulting subset of data, RTs were removed if they fell outside of a 2.5 SD range around the mean naming latency for a given participant within each language. The cleaning procedure resulted in the removal of 5.4% of correct trials. Comprehension questions followed filler sentences only. The mean comprehension question accuracy was 83% and did not depend on the language of the filler trial [*t*(26) = 0.67; *p* > 0.05; *r*^2^ = 0.02].

Accuracies and RTs (see Table 2) for word naming were submitted to 2 × 2 × 2 repeated measures ANOVAs with switching (switch or non-switch trial), language (English or Spanish), and cognate status (cognate or non-cognate) as within-subjects factors. For naming accuracy, there was a small but significant main effect of cognate status such that cognates were named slightly less accurately than controls [cognates: *M* = 0.95; non-cognates: *M* = 0.96; *F*_1_(1,26) = 6.57, *p* < 0.05, $\eta_{p}^{2}=0.202$, *F*_2_(1,252) = 2.43, *p* > 0.05, $\eta_{p}^{2}=0.01$]. No other effects were significant (see Table 3 for detailed results). Like the objective measures of language proficiency, the analysis of naming accuracy suggests that the Spanish-English bilinguals who participated in the study were highly proficient in each language.

**Table 2:** **Mixed-language results**.

Measure	Word type	English				Spanish			
		Non-switch		Switch		Non-switch		Switch	
		M	SD	M	SD	M	SD	M	SD
Naming latency	Cognate	700	120	705	119	699	131	707	143
	Non-cognate	710	112	715	125	719	151	721	145
	Cognate effect	−10		−10		−20		−14	
Accuracy	Cognate	0.94	0.12	0.93	0.13	0.96	0.12	0.96	0.11
	Non-cognate	0.95	0.13	0.96	0.12	0.96	0.13	0.96	0.13
	Cognate effect	−0.01		−0.03		0.00		0.00	

*Mean naming latency (in milliseconds) and accuracy in Experiment 1 as a function of language, switching, and cognate status*.

**N* = 27*.

**Table 3:** **Mixed-language ANOVA results**.

Measure	Effect	*F*_1_			*F*_2_		
		Test	*p*	$\eta_{p}^{2}$	Test	*p*	$\eta_{p}^{2}$
Naming Latency	Cognate status	*F*(1,26) = 7.53*	<0.05	0.22	*F*(1,252) = 8.69*	<0.05	0.03
	Switching	*F*(1,26) = 2.40	>0.05	0.09	*F*(1,252) = 1.79	>0.05	0.01
	Language	*F*(1,26) < 1	>0.05	<0.01	*F*(1,252) < 1	>0.05	0.01
	Switching × language	*F*(1,26) < 1	>0.05	<0.01	*F*(1,252) < 1	>0.05	0.01
	Switching × cognate status	*F*(1,26) < 1	>0.05	<0.01	*F*(1,252) < 1	>0.05	<0.01
	Language × cognate status	*F*(1,26) = 1.27	>0.05	0.05	*F*(1,252) = 1.06	>0.05	<0.01
	Switching × language × cognate status	*F*(1,26) < 1	>0.05	<0.01	*F*(1,252) < 1	>0.05	<0.01
Accuracy	Cognate status	*F*(1,26) = 6.57*	<0.05	0.202	*F*(1,252) = 2.43	>0.05	0.01
	Switching	*F*(1,26) < 1	>0.05	0.02	*F*(1,252) < 1	>0.05	<0.01
	Language	*F*(1,26) = 3.19	>0.05	0.11	*F*(1,252) = 3.07	>0.05	0.01
	Switching × language	*F*(1,26) < 1	>0.05	0.03	*F*(1,252) < 1	>0.05	<0.01
	Switching × cognate status	*F*(1,26) = 1.42	>0.05	0.05	*F*(1,252) < 1	>0.05	<0.01
	Language × cognate status	*F*(1,26) = 3.44	>0.05	0.12	*F*(1,252) < 1	>0.05	<0.01
	Switching × language × cognate status	*F*(1,26) = 2.78	>0.05	0.1	*F*(1,252) = 1.84	>0.05	<0.01

*Results of 2 × 2 × 2 repeated measures ANOVAs on naming latency and accuracy for Experiment 1*.

For RT data, there was a main effect of cognate status, with cognates significantly faster than non-cognate controls [cognates = 703 ms vs. non-cognates = 716 ms; *F*_1_(1,26) = 7.53, *p* < 0.05, $\eta_{p}^{2}=0.22$, *F*_2_(1,252) = 8.69, *p* < 0.05, $\eta_{p}^{2}=0.03$]. No other effects were significant (see Table 3 for detailed results).

### Discussion

There were three important results in Experiment 1. First, there was no evidence to suggest that it is costly to switch between languages; words were named as quickly in sentence contexts that followed a language switch as sentence contexts that did not switch. Second, there was a cognate effect such that cognates were named faster than controls, suggesting that both languages were activated in parallel. Third, the magnitude of the cognate effect was not influenced by the broader context of sentence presentation; the magnitude of the cognate effect was independent of language switching across sentences and also independent of the language of naming. The absence of language-specific differences in English and Spanish is likely to be due to the fact that the bilinguals in this experiment were relatively balanced across the two languages. The absence of switch costs and relatively constant effects of cognate status are consistent with the predictions of the BIA+ model. The implications of these findings will be examined in more detail in the General Discussion.

A limitation of this experiment is that it cannot rule out an influence of the mixed presentation block on the effects of switching and cognate facilitation. A mixed context might result in an overall cost of mixing, overshadowing costs of sentence-to-sentence switching. Additionally, the relative activation of the two languages might already be at its peak in a mixed-language block, maximizing the magnitude of cognate facilitation. To overcome this limitation, a second experiment was conducted in which the same materials were presented in separate lists blocked by language.

## Experiment 2: Blocking the Language of Sentence Context

The results of Experiment 1 suggest that there is no cost to inter-sentential language switching when participants are required to name words embedded in sentences, and that inter-sentential switching does not influence the magnitude of language co-activation. However, a mixed-language context is in some sense a more difficult task because it requires the constant maintenance of activation of two languages, and this maintenance may induce a global mixing cost. If the global mixing cost is evident on both switched and non-switch trials, then trial-by-trial switch costs may be overshadowed, and the heightened activation of both languages may result in a maximal effect of cognate status, which may be masking effects of trial-to-trial switching. To test this hypothesis, a new set of bilingual participants, closely matched to those in Experiment 1, read the same set of materials in a blocked language paradigm. By comparing the results of the blocked language experiment to those of Experiment 1, the costs of language mixing can be investigated. If language mixing is costly and results in a maximal cognate effect, then a blocked language presentation should result in relatively faster naming times for target words and a smaller magnitude of the cognate facilitation effect. The BIA+ model predicts that the task context itself has no influence on the earliest stages of lexical access, so little effect language mixing would be predicted. Likewise the magnitude of the cognate effect should not depend on whether the targets were presented in the mixed block or in the single-language block.

### Methods

#### Participants

Twenty-six participants were recruited for the blocked experiment. Fifteen participants were students at Penn State University and 11 were students at the University of Texas, El Paso. Participants from Penn State University were paid $10 for their participation, and the participants from UTEP were recruited from the subject pool. All subjects gave informed consent, and the procedures had the approval of the Institutional Review Board of the Pennsylvania State University. All of the bilinguals were native Spanish speakers for whom English was the L2, though age of second language acquisition varied. Eleven participants reported speaking both Spanish and English at home (reported age of acquisition in years: *M* = 5.4), and 15 participants reported speaking only Spanish at home (reported age of acquisition in years: *M* = 7.0). The participants in Experiment 2 completed the same linguistic and cognitive tasks as those in Experiment 1. The full set of participant characteristics from Experiment 2 (in addition to the characteristics of participants from Experiment 1) is shown in Table 1.

The participants from Experiment 2 were compared to participants from Experiment 1 to assess how well the two groups were matched on the participant characteristic measures. The two groups differed slightly in mean age with participants in Experiment 1 being slightly older (*M* = 26.0) than participants in Experiment 2 [*M* = 21.92; *F*(1,50) = 5.32, *p* < 0.05, $\eta_{p}^{2}=0.10$]. The two groups marginally differed in their working memory capacity such that participants in Experiment 1 had a numerically lower Operation-Span score (*M* = 34.67) than participants in Experiment 2 [*M* = 40.35; *F*(1,44) = 3.60, *p* = 0.06, $\eta_{p}^{2}=0.07$]. The two groups did not differ in executive function as measured by the Simon Task [Experiment 1: *M* = 46 ms; Experiment 2: *M* = 47 ms; *F*(1,46) < 1; *p* > 0.05, $\eta_{p}^{2}<0.01$]. Critically, there was no evidence that the two groups differed in measures of subjective or objective language proficiency (comprehension question performance on the main task, language self-ratings, grammar task performance), nor was there a difference in the magnitude of parallel activation as measured by the English picture naming task (all *F* < 1, all *p* > 0.05, all $\eta_{p}^{2}<0.02$).

#### Materials

The materials (including critical words and sentences) were the same as in Experiment 1. The only difference was that the sentences were presented in separate language blocks, rather than in alternating runs.

#### Procedure

The procedure of Experiment 2 was similar to the one described for Experiment 1 with the following differences. During the first session of the blocked experiment, participants completed one block of the main task (English or Spanish; order counterbalanced across participants). Following the main task, they completed all other tasks (O-Span, Simon, English picture naming). During the second session of the task, participants completed the second block of the main task and the English and Spanish grammar tasks. All other procedural information was the same as in Experiment 1^3^.

### Results

Reaction time data were coded for accuracy and cleaned using the procedure outlined in Experiment 1. The cleaning procedure resulted in the removal of 3.1% of correct trials. As in Experiment 1, comprehension questions followed filler sentences only. The mean comprehension question accuracy was 84% and did not depend on the language of the sentence [*t*(25) = 1.11; *p* > 0.05; *r*^2^ = 0.05]. This value was similar to the 83% accuracy on comprehension questions reported for Experiment 1.

Accuracy and RT data (see Table 4) from the blocked design were submitted to 2 × 2 repeated measures ANOVAs with language (English or Spanish) and cognate status (cognate or non-cognate) as within-subjects factors. For naming accuracy, no main effects or interactions were significant (see Table 5 for detailed results). Analyses on RT data revealed a main effect of cognate status [cognates: *M* = 676 ms; non-cognates: *M* = 691 ms; *F*_1_(1,25) = 17.47, *p* < 0.001, $\eta_{p}^{2}=0.41$, *F*_2_(1,252) = 5.94, *p* < 0.05, $\eta_{p}^{2}=0.02$]. There was a main effect of language by items only [*F*_1_(1,25) = 2.16, *p* > 0.05, $\eta_{p}^{2}=0.08$, *F*_2_(1,252) = 29.75, *p* < 0.05, $\eta_{p}^{2}=0.11$] such that RTs were faster to name English (M = 667 ms) than Spanish (*M* = 700 ms), but there was no interaction between language and cognate status (see Table 5 for detailed Results). The main pattern of results was thus similar to the one reported for Experiment 1.

**Table 4:** **Blocked language results**.

Measure	Word type	English		Spanish	
		*M*	SD	*M*	SD
Naming latency	Cognate	658	137	693	183
	Non-cognate	676	146	706	182
	Cognate effect	−18		−13	
Accuracy	Cognate	0.97	0.04	0.97	0.04
	Non-cognate	0.96	0.05	0.97	0.04
	Cognate effect	0.01		0.00	

*Mean naming latency (in milliseconds) and accuracy in Experiment 2 as a function of language and cognate status*.

**N* = 26*.

**Table 5:** **Blocked language ANOVA results**.

Measure	Effect	*F*_1_			*F*_2_		
		Test	*P*	$\eta_{p}^{2}$	Test	p	$\eta_{p}^{2}$
Naming Latency	Cognate status	*F*(1,25) = 17.47*	<0.001	0.41	*F*(1,252) = 5.94*	<0.05	0.02
	Language	*F*(1,25) = 2.16	>0.05	0.08	*F*(1,252) = 29.75*	<0.05	0.11
	Cognate status × language	*F*(1,25) < 1	>0.05	0.02	*F*(1,252) < 1	>0.05	<0.02
Accuracy	Cognate status	*F*(1,25) < 1	>0.05	<0.01	*F*(1,252) < 1	>0.05	<0.01
	Language	*F*(1,25) < 1	>0.05	<0.01	*F*(1,252) < 1	>0.05	<0.01
	Cognate status × language	*F*(1,25) < 1	>0.05	0.03	*F*(1,252) < 1	>0.05	<0.01

*Results of 2 × 2 repeated measures ANOVAs on naming latency and accuracy for Experiment 2*.

#### Comparing mixed and blocked sentence contexts

To examine effects of language mixing, non-switch trials from Experiment 1 were compared to the blocked data collected in Experiment 2. Accuracy data and naming latency data (see Table 6) were submitted to 2 × 2 × 2 repeated measures ANOVAs with language (English or Spanish) and cognate status (cognate or non-cognate) as within-subjects factors and experiment (mixed or blocked) as a between subjects factor. In the accuracy data, the effect of experiment was significant by items only [*F*_1_(1,51) < 1, *p* > 0.05, $\eta_{p}^{2}<0.01$, *F*_2_(1,252) = 6.83, *p* < 0.05, $\eta_{p}^{2}=0.03$], with the accuracy in the mixed experiment 2% lower (*M* = 0.95) than in the blocked experiment (*M* = 0.97). No other effects were significant (see Table 7 for detailed results).

**Table 6:** **Mixed and blocked group comparison**.

Measure	Group*	Word type	English		Spanish	
			Mean	SD	Mean	SD
Naming latency	Experiment 1: mixed	Cognate	700	120	699	131
		Non-cognate	710	112	719	151
		Cognate effect	−10		−20	
	Experiment 2: blocked	Cognate	658	137	693	183
		Non-cognate	676	146	706	182
		Cognate effect	−18		−13	
Accuracy	Experiment 1: mixed	Cognate	0.94	0.12	0.96	0.12
		Non-cognate	0.95	0.13	0.96	0.13
		Cognate effect	−0.01		0.00	
	Experiment 2: blocked	Cognate	0.97	0.04	0.97	0.04
		Non-cognate	0.96	0.05	0.97	0.04
		Cognate effect	0.01		0.00	

*Comparison of naming latencies (in milliseconds) and accuracies from non-switched trials of Experiment 1 and all trials of Experiment 2 as a function of language and cognate status*.

*N_Mixed_ = 27; N_Blocked_ = 26.

**Table 7:** **Mixed and blocked comparison ANOVAs**.

Measure	Effect	*F*_1_			*F*_2_		
		Test	*p*	$\eta_{p}^{2}$	Test	*p*	$\eta_{p}^{2}$
Naming latency	Experiment	*F*(1,51) < 1	>0.05	0.01	*F*(1,252) = 63.99*	<0.05	0.2
	Cognate Status	*F*(1,51) = 17.09*	<0.001	0.25	*F*(1,252) = 7.23*	<0.05	0.03
	Language	*F*(1,51) = 1.91	>0.05	0.04	*F*(1,252) = 9.53*	<0.05	0.04
	Language and experiment	*F*(1,51) = 1.11	>0.05	0.02	*F*(1,252) = 12.35*	<0.05	0.05
	Experiment in English				*F*(1,252) = 70.06*	<0.001	0.36
	Experiment in Spanish				*F*(1,252) = 9.59*	<0.05	0.07
	Cognate status × experiment	*F*(1,51) < 1	>0.05	<0.01	*F*(1,252) < 1	>0.05	<0.01
	Language × cognate status	*F*(1,51) < 1	>0.05	<0.01	*F*(1,252) < 1	>0.05	<0.01
	Language × cognate status × experiment	*F*(1,51) = 1.15	>0.05	0.02	*F*(1,252) < 1	>0.05	<0.01
Accuracy	Experiment	*F*(1,51) < 1	>0.05	<0.01	*F*(1,252) = 6.83*	<0.05	0.03
	Cognate status	*F*(1,51) < 1	>0.05	0.01	*F*(1,252) <1	>0.05	<0.01
	Language	*F*(1,51) = 1.96	>0.05	0.04	*F*(1,252) = 1.52	>0.05	<0.01
	Language × experiment	*F*(1,51) = 1.44	>0.05	0.03	*F*(1,252) = 2.27	>0.05	0.01
	Cognate status × experiment	*F*(1,51) = 1.14	>0.05	0.02	*F*(1,252) = 1.00	>0.05	<0.01
	Language × cognate status	*F*(1,51) < 1	>0.05	<0.01	*F*(1,252) < 1	>0.05	<0.01
	Language × cognate status × experiment	*F*(1,51) < 1	>0.05	0.01	*F*(1,252) < 1	>0.05	<0.01

*Results of 2 × 2 × 2 repeated measures ANOVAs (and simple effects tests) comparing the results of Experiments 1 and 2*.

In the analysis of RTs, a significant cognate effect emerged with faster naming latencies for cognates (*M* = 687 ms) compared to non-cognate controls [*M* = 703 ms; *F*_1_(1,51) = 17.09, *p* < 0.001, $\eta_{p}^{2}=0.25$, *F*_2_(1,252) = 7.23, *p* < 0.05, $\eta_{p}^{2}=0.03$]. There was also a main effect of language significant only by items [*F*_1_(1,51) = 1.91, *p* > 0.05, $\eta_{p}^{2}=0.04$, *F*_2_(1,252) = 9.53, *p* < 0.05, $\eta_{p}^{2}=0.04$] with RTs for English naming faster (*M* = 686 ms) than Spanish naming (*M* = 704 ms). The effect of experiment was significant by items only [*F*_1_(1,51) < 1, *p* > 0.05, $\eta_{p}^{2}=0.01$, *F*_2_(1,252) = 63.99, *p* < 0.05, $\eta_{p}^{2}=0.20$], with RTs in the blocked context faster (*M* = 683 ms) than the mixed context (*M* = 706 ms). There was also a significant interaction between language and experiment in the items analysis only [*F*_1_(1,51) = 1.11, *p* > 0.05, $\eta_{p}^{2}=0.02$, *F*_2_(1,252) = 12.35, *p* < 0.05, $\eta_{p}^{2}=0.05$], suggesting a larger mixing cost for English (mixed context: *M* = 705 ms; blocked context: *M* = 667 ms; *F*_2_ = 70.06, *p* < 0.001, $\eta_{p}^{2}=0.36$) than for Spanish (mixed context: *M* = 709 ms; blocked context: *M* = 700 ms; *F*_2_ = 9.59, *p* < 0.05, $\eta_{p}^{2}=0.07$). No other interactions approached significant (see Table 7 for detailed results). Other than the interaction between mixing costs and language in the item analysis, there was little evidence overall for an effect of language mixing on overall RT or, critically, on the presence or magnitude of the cognate effect. The cognate effect was robust under all conditions.

### Discussion

The results of Experiment 2 rule out the explanation that a language mixing environment has additional processing costs above and beyond what can be observed in a comparison between switch and non-switch trials. When the mixed-language results were compared to those of the blocked language presentation, there was only a difference in naming times for English and only in the item analysis, suggesting that there was not a general or reliable effect of language mixing. Furthermore, a cognate effect was present in both the mixed-language presentation and the blocked language presentation. Critically, the magnitude of the cognate effect did not change between the two methods of presentation, suggesting that the broader language context had little or no influence on the degree of parallel activation of the two languages. Despite potentially salient contextual information (e.g., blocked language vs. mixed-language sentence context; switch vs. non-switch sentence context) in the input, the bilinguals in these two experiments did not appear to exploit contextual information, and the degree of activation of their two languages remained unchanged across Experiments 1 and 2. The experiments also suggest that language mixing and language switching do not incur a cost for words embedded in sentences. These results are consistent with the counterintuitive prediction of the BIA+ model that the non-selectivity of bilingual lexical access cannot be overcome by expectations or contextual cues.

## General Discussion

The current study tested the counterintuitive prediction made by BIA+ model that sentence context and predictability of the language of the sentence context play a marginal, if any, role in lexical access. Robust cognate facilitation was measured during the production of words embedded in unilingual sentence contexts. The magnitude of the effect did not depend on the broader context of presentation (switching, mixing, or blocking), nor did it depend on the language of the sentence. However, participants did successfully name target words aloud in the appropriate language with relatively few errors, suggesting the existence of a control mechanism that can restrict language production to a single-language. Both languages were apparently activated despite the intention to use one language alone, and the presence of potential cues to the target language did not function to direct language selection during word recognition. Inhibitory processes are frequently implicated as a potential mechanism for successful bilingual language comprehension and production, and such an account predicts costs to language switching. However, there was no measureable cost to the requirement to switch or mix languages present in the current study. Both of these results are consistent with the BIA+ model of word recognition (Dijkstra and van Heuven, 2002) and have implications for the role of cognitive control during bilingual language selection.

### Parallel cross-language activation

Robust cognate facilitation independent of the presence of context is in line with many previous results demonstrating that bilingual word recognition is non-selective with regard to language (Schwartz and Kroll, 2006; Duyck et al., 2007; Libben and Titone, 2009; Van Assche et al., 2010; Titone et al., 2011). The present study extends the results of the previous studies in a number of ways. Like past studies, the present experiments reveal cognate facilitation even when bilinguals know in advance the language in which the target words will appear. Unlike past studies, the experiments reported here show that switches in the language of the sentence context have virtually no effect on either the absolute time to process target words or on the magnitude of cognate facilitation. Despite the presence of predictable and potentially informative cues encoded in the language input, bilinguals activated both languages in parallel and to the same degree.

The present results conflict with studies that find that additional layers of context can decrease the activation of the unintended language. For example, if a sentence contains a highly biased interpretation, then the cognate effect is reduced to the point at which it is no longer observable (Schwartz and Kroll, 2006; Libben and Titone, 2009; but see Van Assche et al., 2010). If the language context (i.e., switched or non-switched trial, mixed or blocked presentation) could function as a cue to enable selection of the target language, then the magnitude of the cognate effect should be larger in mixed than in blocked contexts and larger following switched than non-switched contexts. The data presented here provided little support for these predictions. There was no difference in the magnitude of cognate facilitation in switched vs. non-switched sentence contexts. Moreover, the effect of mixed-language presentation was significant only in the item analysis, where there was a suggestion that there was a cost to language mixing that was larger for naming words in English than in Spanish. These results do not rule out the possibility that other types of contextual or language-specific information may function as informative language cues either, nor do they rule out the possibility that the language context manipulation investigated here can function as an effective language cue when paired with other layers of context (e.g., semantic constraint). These results do suggest that the presence of one language alone does not suffice to reduce the activation of lexical candidates in both languages.

A possible alternative account for the observed pattern of results is that the cognate effect does not reflect language co-activation but is rather a relative frequency effect. In theory, because cognates are present in both languages, they come to have a higher functional frequency of use compared to non-cognate control words, which would elicit comparably facilitated processing. Electrophysiological evidence suggests that both cognate status and word frequency affect an N400-like component with a similar time-course of processing, indicating that cognate effects and word frequency effects may share the same locus (e.g., Strijkers et al., 2010). If the cognate effect maps onto a difference in relative frequency, then the magnitude of the cognate effect might be independent of the linguistic context in which they are presented.

While plausible, the relative frequency hypothesis cannot be the sole locus of the cognate facilitation effect. Cognate facilitation can be modulated via the degree of form overlap (orthographic or phonological overlap; e.g., Schwartz et al., 2007; Van Assche et al., 2010), suggesting that some form of language co-activation takes place during the processing of this type of stimuli. Additionally, while the cognate and word frequency effects may impact similar N400-like components, the scalp distributions of the two effects have not, to our knowledge, been directly compared. Furthermore, Strijkers et al. (2010) did obtain an interaction between cognate status and word frequency, suggesting independent contributions of each manipulation, though the interaction occurred later in the time-course of processing. In any case, the origin of any cognate frequency effect must be due to the presence of two languages operating within a single cognitive system, as monolingual speakers of each language show no evidence of cognate facilitation.

The present results instead demonstrate the stability of language co-activation that is measured by cognates, in contrast to other language-ambiguous stimuli such as homographs. Homograph inhibition is more variable than cognate facilitation, as it depends on the construction of the stimulus set and on task. For example, homograph effects may be too weak to observe in a lexical decision task unless non-target-language fillers are also present, and homograph inhibition can become facilitation if bilinguals can make a “yes” lexical decision to both of their languages (e.g., Dijkstra et al., 1998). This variability suggests that homographs may be more sensitive to contextual manipulations than cognates. Research utilizing homographs as an index of parallel activation indicates that may be able to utilize global task contexts to “zoom in” to the L2 and ignore influence from the L1 (e.g., Elston-Güttler et al., 2005; Paulmann and Elston-Güttler, 2006; Elston-Guttler and Gunter, 2009). It will remain to be seen whether the manipulation of the language of the sentence context differentially affects the processing of homographs that may be more vulnerable to the conditions of presentation than cognates.

### How far into a sentence does the language of the sentence affect lexical access?

A possible objection to the interpretation we have offered is that we used a lexically focused behavioral task, potentially reflecting the processing of only the target word independent of the sentence or task context in which it is embedded. However, participants were able to respond to the comprehension questions in the main task with high accuracy, suggesting that they were successfully constructing the meaning of the sentence. Research in the monolingual domain shows that semantic and discourse level contextual information is maintained during sentence processing and is utilized during the processing of visually presented words embedded within a sentence, even when an overt task-decision (e.g., naming) is required on a single word. For example, named targets become facilitated following a congruent semantic context. The facilitation can be observed in the absence of lexically associated words, and it is not observed when sentences are presented with scrambled word order, suggesting that the effects are not due to simple lexical priming (Simpson et al., 1989; Simpson and Krueger, 1991). Instead the effects can be attributed to activation and integration of higher level contextual information. Discourse level information is similarly maintained and activated during the processing of words in context (O’Brien et al., 1986; Binder and Morris, 1995), and similar effects have been found in bilingual speakers (e.g., Schwartz and Kroll, 2006; Van Hell and de Groot, 2008). In the context of the present study, it appears that message-level information encoded in the sentence may act to reduce language switching costs, but that this message-level activation does not influence the parallel activation of each of the languages.

### Language switch costs

A second finding of the study involves costs related to language switching. Despite substantial psycholinguistic evidence to suggest that switching languages incurs a processing cost for bilingual speakers, no such cost was observed for the naming of target words within sentence contexts that switched inter-sententially. The current investigation bridges the gap between the observational and experimental studies on code-switching, demonstrating that language switching and language mixing may not incur costs. These results are consistent with those of other studies on the comprehension of code-switched sentences. For instance, Ibáñez et al. (2010) demonstrated that when bilinguals read sentences for comprehension, there is no evidence for a cost to switching between languages inter-sententially. Likewise, Guzzardo Tamargo (2012) found no evidence for a switch cost at the site of the switch when bilinguals read intra-sententially code-switched sentences. In each of these studies, switch costs did arise when a non-comprehension-based task was added to the design. Ibáñez et al. observed a cost when bilinguals were burdened with the task of remembering the sentence in order to repeat it later and Guzzardo Tamargo observed a switch cost when participants were asked to make metalinguistic judgments about the sentences. This suggests that during normal reading bilinguals may recruit memory resources to negotiate switching between languages. When these resources are taxed, switch costs become evident. Taken together, such results and those reported here indicate that in normal circumstances, language switching does not incur a cost so long as there is sufficient linguistic context (i.e., a sentence context) available. Hence, a sentence context may provide information that can help bilinguals overcome the inhibition that is applied to a language in order to suppress it during the use of the intended language.

It is also possible that there are indeed switch costs in sentence processing but that those costs diminish at some point into the sentence. In the present study, all critical targets were presented in the middle of each sentence, so that by the time the target word appeared, the switch costs may have dissipated. If switching costs were a purely local effect, then costs should manifest only at the site of a switch. Because the present methodology included a measure of naming latency on only the target word, costs at the site of the switch cannot be analyzed. However, Ibáñez et al. (2010) measured inter-sentential switch costs at initial region of sentences in a self-paced reading task and they found no evidence for a cost to switching between languages, suggesting that there is no local cost to inter-sentential switching. Although inter-sentential code-switching in naturalistic settings is unlikely to follow an alternating runs sequence, the finding of no switch costs imposed by the artificial switching sequence suggests that under conditions in which bilinguals themselves control the order of switches, there are likely to be even fewer processing costs.

A question regarding the present investigation is whether the lack of switch costs is related to the fact that language switches were highly predictable. In Experiment 1, the language of the sentence context switched after every two sentences. In theory, alternating runs could have induced a strategy of processing that eliminated switch costs, particularly if those costs were small because the point of measurement was distant from site of the language switch. Indeed, studies in the task-switching literature show that recovery from a task switch occurs very quickly in cases where switches are predictable but recovery is attenuated in cases where switching is random (e.g., Monsell et al., 2003). Critically, costs are still present and significant in cases of predictable switching (e.g., MacNamara et al., 1968; Meuter and Allport, 1999; Monsell et al., 2003; Gollan and Ferreira, 2009). In the context of the present investigation, highly predictable switches may have negated local (i.e., word-to-word) switch costs, but costs generated at a more global level (i.e., switching the language of the sentence context) should have been observable. In the present experiments, we used sentences in which target words were not highly constrained as a means to identify the role of the language of the sentence context itself. In actual discourse and natural code-switching, prediction at both the local and global levels plays a crucial role (e.g., Federmeier, 2007). In future research it will be of interest to determine whether the absence of switch costs induced by alternating the language of sentence context will interact with or be independent of linguistic features of the sentence.

Overall, the present results regarding parallel activation and language switching are entirely consistent with predictions made by BIA+ model of word recognition (Dijkstra and van Heuven, 2002). In the BIA+ model, the word recognition portion of the system provides feed-forward activation to a task schema with no feedback or bi-directional connections. Hence, the model predicts that there should be no influence of contexts (such as task demands) on the non-selectivity of the word recognition system, so the magnitude of the cognate effect is predicted to be independent of the language context (e.g., language mixing relative to language blocking). According to BIA+, word recognition proceeds as a primarily bottom-up process, with few top-down influences. The model also predicts that in the present design there should be no costs associated with switching languages because the language of the word presentation stays the same within a unilingual sentence. Evidence for both of these predictions was found in the present set of experiments. The prediction that there should be no switch costs within a sentence hinges on the presence of a sentence context (i.e., previous words in a sentence function to negate the presence of a switch). However, within the framework of BIA+, the sentence context does not function in a top-down manner to alter the relative activations of words in each language as alternative accounts might predict. It is simply a byproduct of the fact that the task-decision system does not have to switch languages in order to process the target word. Future studies should disentangle the locus of the reduced switch costs in sentence context. Are reduced switch costs simply a side-effect of the target words following words of the same language, or is context influencing the degree of inhibition through a more top-down and proactive process?

The present results also contribute to the extant knowledge about inhibitory control in bilingual language use. A recent debate has surfaced as to the scope and time-course of recovery from inhibition of the unintended language. If inhibition of a language is applied to singular words (e.g., inhibit *table* while reading the Spanish word *mesa*, which means *table* in English), then inhibition may be relatively short-lived and easy to overcome. In contrast, if an entire language is inhibited, then one might expect inhibition to be more long lasting and persist far into the processing (e.g., far into the next sentence) following a language switch. These two possibilities are not mutually exclusive, and it may be overly simplistic to confound scope and time-course. However, the results here show that there is seemingly no inhibition to overcome by the time the participant reaches the middle of a sentence following a language switch because there are no observable costs. This suggests that inhibition (at least in comprehension) is short-lived and may not be applied to an entire language as a whole.

## Conclusion

This study bridges a gap between some somewhat conflicting findings across two disciplines of language science. Linguistic studies on code-switching demonstrate that bilinguals can switch languages seamlessly in a manner that follows grammatical patterns or rules. Yet, at the same time, psycholinguistic evidence from language switching experiments suggests that a language switch comes with an observable cost. Our results suggest that when bilinguals read sentences in either of their known languages, the language not in use is not only active, but it is active to the same extent whether it was used mere seconds ago (i.e., language switching) or several days ago (i.e., blocked by language). Counter intuitively, it appears that the mixing sentences from two languages in a bilingual production task does not change the activation state of the language currently not in use. This suggests that context does not influence word recognition, in line with an influential model of bilingual word recognition. If it is true that the language context does not increase or decrease the degree of language co-activation and there is no cost of switching languages, it is not particularly surprising that bilinguals exploit the ability to code-switch in real life contexts.

## Conflict of Interest Statement

The authors declare that the research was conducted in the absence of any commercial or financial relationships that could be construed as a potential conflict of interest.

## Supplementary Material

The Supplementary Material for this article can be found online at http://www.frontiersin.org/Language_Sciences/10.3389/fpsyg.2013.00278/abstract

## Footnotes

^1^The Spanish and English grammar tests were not originally designed for explicit comparison of the relative proficiency of the two languages. The comparison is shown here to illustrate the balance of the two languages.

^2^Form overlap was calculated as a ratio based on a measure of graphemic overlap developed by Van Orden (1987).

^3^Changes in the order of tasks between Experiment 1 and Experiment 2 were made for logistical reasons. In Experiment 1, participants were required to complete the experiment proper along with a battery of linguistic and cognitive tasks. To avoid participant fatigue, it was decided to divide the experimental session into two sessions. The experiment proper was administered in session 1 and the remaining tasks in session 2. Experiment 2 necessarily had a breaking point between the two language blocks. The blocks were presented across two sessions to preclude effects of block ordering (e.g., seeing L1 after naming the L2) that could result in unintended inhibitory effects during task performance (e.g., Misra et al., 2012). To avoid participant fatigue, the battery of tasks was also divided up between the two sessions (and was always presented after the experiment proper).
