# Supplementary material for: When Language Switching has No Apparent Cost: Lexical Access in Sentence Context
Source: Front Psychol. 2013 May 30;4:278. doi: 10.3389/fpsyg.2013.00278 (PMC3668438; doi:10.3389/fpsyg.2013.00278)
Supplement: Supplementary file 1 [file 45237_Gullifer_Presentation1.PDF]

When language switching has no apparent cost: Lexical access in sentence context

Jason W. Gullifer[1,3], Judith F. Kroll[1,3], & Paola E. Dussias[2,3]

[1] Department of Psychology, Pennsylvania State University, University Park, PA, USA

[2] Department of Spanish, Italian, and Portuguese, Pennsylvania State University, University Park, PA, USA

[3] Center for Language Science, Pennsylvania State University, University Park, PA, USA

Direct correspondence to:

Jason Gullifer
Pennsylvania State University
Department of Psychology
Moore Building
University Park, PA 16802 USA
jwg20@psu.edu

Appendix A

English cognate stimuli. For each target word, frequency of occurrence (Brysbaert & New , 2009; Kuçera & Francis, 1986), an orthographic similarity ratio based on Van Orden (1987), and number of letters are indicated.

Cognate	Word Frequency (Kuçera & Francis)	Word Frequency (SUBTLEX)	Orthographic Similarity	Orthographic Length
bus	34	74.18	1.00	3
general	497	115.39	1.00	7
colleagues	23	6.51	0.72	10
garage	21	27.22	0.81	6
cable	7	21.73	1.00	5
project	93	37.39	0.64	7
camera	36	57	0.75	6
tourists	12	5.41	0.79	8
giraffe	0	1.49	0.36	7
reporter	20	20.39	0.83	8
plate	22	25.65	0.70	5
pirate	4	7.35	0.77	6
pipe	20	19.39	0.71	4
plant	125	27.61	0.77	5
professor	57	69.57	0.76	9
statue	17	10.59	0.48	6
client	15	53.24	0.78	6
cobra	3	3.33	1.00	5
cube	1	2.98	0.66	4
organizer	3	0.43	0.83	9
violin	11	4.75	0.82	6
circle	60	21.51	0.44	6
pistol	27	10.06	0.78	6
official	75	24	0.93	8
broccoli	1	2.27	0.77	8
caramels	1	0.18	0.91	8
family	331	354.25	0.73	6
president	382	140.67	0.80	9
student	131	43.04	0.34	7
receptionist	5	1.9	0.73	12
sofa	6	5.86	0.66	4
biologist	2	1.25	0.54	9

problems	247	77.08	0.91	8
computer	13	59.04	0.62	8
detective	52	61.12	1.00	9
athlete	9	4.61	0.64	7
composer	31	2.49	0.81	8
colonel	37	96.25	0.78	7
patient	86	63.65	0.64	7
hamburger	6	7.78	0.66	9
capitals	4	0.53	0.91	8
soup	16	25.2	0.53	4
vendor	1	1.27	0.92	6
decision	119	55.06	0.88	8
rat	6	32.61	0.82	3
sweater	14	13.8	0.69	7
engineer	42	11.69	0.38	8
baby	62	509.37	0.33	4
institute	50	3.25	0.79	9
tank	12	25.61	0.58	4
director	101	35.96	1.00	8
strategy	22	9.45	0.43	8
coffee	78	144.53	0.31	6
cathedral	8	3.73	0.90	9
telephone	76	32.37	0.56	9
carpenter	6	6	0.68	9
presenter	1	0.39	0.82	9
artist	57	28.63	0.78	6
cereal	17	6.35	1.00	6
dynamite	5	10.1	0.62	8
authorities	39	12.78	0.72	11
members	325	24	0.67	7
antelope	7	0.98	0.87	8
kangaroo	0	2.31	0.46	8

English non-cognate stimuli. For each target word, frequency of occurrence (Brysbaert & New , 2009; Kuçera & Francis, 1986), an orthographic similarity ratio based on Van Orden (1987), and number of letters are indicated.

Control	Word Frequency (Kuçera & Francis)	Word Frequency (SUBTLEX)	Orthographic Similarity	Orthographic Length

hairspray	0	0	0.05	9
deed	8	9.31	0.03	7
notebook	2	4.61	0.14	8
handling	38	11.35	0.13	8
spark	12	6.27	0.28	5
neighborhood	58	36.69	0.06	12
spring	127	31.31	0.20	6
wound	28	26.53	0.06	5
steak	10	16.24	0.28	5
fisherwoman	0	0	0.09	11
tower	13	22.84	0.56	5
report	174	108	0.06	8
dog	75	192.84	0.06	3
iron	43	17.94	0.20	4
wash	37	40.73	0.05	4
foam	37	3.51	0.14	4
dessert	7	14.02	0.17	7
ribbon	12	5.06	0.07	6
childhood	50	14.18	0.04	9
printer	3	2.1	0.20	7
zipper	1	2.82	0.06	6
punishment	21	13.43	0.07	10
tie	23	44.43	0.04	3
workroom	0	0.22	0.05	6
referee	1	3.59	0.07	7
hair	148	153.55	0.05	4
boys	143	224.16	0.27	4
friend	133	419.29	0.06	6
foreigners	13	3.2	0.36	10
rangers	2	4.78	0.28	7
wall	160	70.69	0.05	4
lamb	7	10.63	0.07	4
soul	47	76.96	0.08	4
ladder	19	9.25	0.21	6
parade	25	12.88	0.05	6
ostrich	0	0.94	0.21	7
freezer	1	5.16	0.19	7
scale	60	9.51	0.24	5
hole	58	58.22	0.07	4
stepbrother	0	0.31	0.28	11
beggar	2	2.47	0.07	6
loin	1	0.35	0.51	4
survey	37	4.45	0.08	6

party	216	233.14	0.08	5
traveler	8	3.02	0.15	8
screen	48	23.39	0.05	6
growth	155	6.45	0.06	6
cave	9	13.98	0.55	4
potato	15	11.29	0.28	6
flour	8	3.16	0.06	5
performance	122	21.82	0.07	11
manager	88	39.96	0.14	7
beauty	71	48.24	0.43	6
elevator	12	24.41	0.31	8
horse	117	92.88	0.05	5
socks	7	18.27	0.23	5
librarian	5	2.88	0.29	9
blind	47	45.82	0.07	5
peaches	1	4.08	0.24	7
plums	1	1.04	0.26	5
guests	62	25.71	0.30	6
building	160	99.57	0.14	8
puppy	1	11.45	0.03	5
elves	1	3.49	0.35	5

Spanish cognate stimuli. For each target word, frequency of occurrence (Alameda & Cuetos, 1995; Sebastián-Gallés, Cuetos, Martí, Carreiras, 2000), an orthographic similarity ratio based on Van Orden (1987), and number of letters are indicated.

Cognate	Word Frequency (Alameda and Cuetos)	Word Frequency (LEXESP)	Orthographic Similarity	Orthographic Length
problemas	279	0	0.91	9
computadora	9	5.36	0.62	11
detective	12	8.21	1.00	9
atleta	8	8.57	0.64	6
compositor	4	1.79	0.81	10
coronel	6	40.18	0.78	7
paciente	52	47.86	0.64	8
hamburguesa	3	1.25	0.66	11
capitales	13	0	0.91	9
sopa	31	15	0.53	4
vendedor	21	8.93	0.92	8

decisión	140	91.43	0.88	8
rata	34	12.5	0.82	4
suéter	6	1.25	0.69	6
ingeniero	70	20.36	0.38	9
bebé	30	16.96	0.33	4
instituto	84	12.32	0.79	9
tanque	12	3.93	0.58	6
director	173	133.21	1.00	8
estrategia	54	41.61	0.43	10
café	210	77.5	0.31	4
catedral	58	20.54	0.90	8
teléfono	186	80.18	0.56	8
carpintero	12	2.32	0.68	10
presentador	3	1.25	0.82	11
artista	102	34.82	0.78	7
cereal	1	1.43	1.00	6
dinamita	2	2.14	0.62	8
autoridades	37	0	0.72	11
miembros	140	0	0.67	8
antílope	1	0.71	0.87	8
canguro	2	0.36	0.46	7
bus	2	0.54	1.00	3
general	632	277.14	1.00	7
colegas	56	0	0.72	7
garaje	22	9.82	0.81	6
cable	16	13.93	1.00	5
proyecto	155	106.25	0.64	8
cámara	114	39.82	0.75	6
turistas	51	0	0.79	8
jirafa	2	1.07	0.36	6
reportero	3	2.32	0.83	9
plato	85	30.89	0.70	5
pirata	12	5.54	0.77	6
pipa	38	14.46	0.71	4
planta	89	38.39	0.77	6
profesora	15	9.64	0.76	9
estatua	36	14.11	0.48	7
cliente	40	21.61	0.78	7
cobra	37	12.5	1.00	5
cubo	26	6.07	0.66	4
organizador	4	3.57	0.83	11
violín	17	5.18	0.82	6
círculo	59	26.43	0.44	7

pistola	50	26.79	0.78	7
oficial	118	79.29	0.93	7
brócoli	1	0	0.77	7
caramelos	15	5	0.91	9
familia	495	210	0.73	7
presidente	138	251.25	0.80	10
estudiante	37	14.82	0.34	10
recepcionista	3	0	0.73	13
sofá	67	17.68	0.66	4
bióloga	1	0.36	0.54	7

Spanish non-cognate stimuli. For each target word, frequency of occurrence (Alameda & Cuetos, 1995; Sebastián-Gallés, Cuetos, Martí, Carreiras, 2000), an orthographic similarity ratio based on Van Orden (1987), and number of letters are indicated.

Control	Word Frequency (Alameda and Cuetos)	Word Frequency (LEXESP)	Orthographic Similarity	Orthographic Length
alma	329	105.89	0.08	4
escalerilla	9	2.68	0.21	11
cabalgata	8	2.5	0.05	9
avestruz	8	2.32	0.21	8
congelador	4	1.07	0.19	10
báscula	7	1.61	0.24	7
agujero	53	21.25	0.07	7
hermanastro	3	0.71	0.28	11
mendigo	13	4.46	0.07	7
lomo	31	7.86	0.51	4
encuesta	21	15.89	0.08	8
fiesta	140	63.57	0.08	6
viajero	33	32.32	0.15	7
biombo	5	2.86	0.05	6
crecimiento	70	49.29	0.06	11
cueva	29	10	0.55	5
papa	84	35.89	0.28	4
harina	12	7.32	0.06	6
actuación	53	42.5	0.07	9
encargado	55	28.04	0.14	9
belleza	212	72.68	0.43	7
ascensor	55	20.54	0.31	8

caballo	187	63.21	0.05	7
calcetines	26	8.39	0.23	10
bibliotecario	4	0	0.29	13
ciegos	73	30.89	0.07	5
duraznos	1	0	0.24	8
ciruelas	3	0.89	0.26	8
huéspedes	37	0	0.30	9
edificio	141	58.57	0.14	8
cachorro	1	0.18	0.03	7
duendes	1	0	0.35	7
laca	2	2.14	0.05	4
escritura	73	25	0.03	9
cuaderno	55	14.11	0.14	8
manejo	24	9.11	0.13	6
chispa	19	7.14	0.28	6
barrio	161	74.11	0.06	6
primavera	114	43.93	0.20	9
herida	52	22.86	0.06	6
bistec	2	0.71	0.28	6
pescadora	1	0.18	0.09	9
torre	85	22.32	0.56	5
folio	13	3.21	0.06	5
perro	38	60.54	0.06	5
hierro	89	20.18	0.20	6
lavado	13	5.36	0.05	6
espuma	36	12.86	0.14	6
postre	41	7.5	0.17	6
cinta	37	17.68	0.07	5
niñez	26	9.29	0.04	5
impresora	4	0.89	0.20	9
bragueta	18	4.82	0.06	8
castigo	59	22.86	0.07	7
corbata	51	17.14	0.04	7
despacho	118	58.75	0.05	8
arbitro	2	0	0.07	7
cabellera	15	7.5	0.05	9
niños	497	0	0.27	5
amiga	136	52.14	0.06	5
extranjeros	40	0	0.36	11
guardabosques	1	0.18	0.28	13
muro	72	26.79	0.05	4
cabrito	1	1.96	0.07	7

Appendix B

Full set of English and Spanish sentence stimuli. Target cognates and controls are indicated in bold.

Spanish		English	
Cognate	Control	Cognate	Control
Marcos le colocó unas calcomanías que compró en la tienda al **bus** durante el fin de semana.	La bailarina le puso la tapa que había perdido el día anterior a la **laca** después de maquillarse.	The man who was returning from the dinner drove the **bus** with the teachers from his school.	The stylist who owns a store downtown sells **hairspray** and other products for hair.
El científico le dio el plan que había ideado al **general** durante la reunión.	El escribano le puso los acentos que había visto que faltaban a la **escritura** de la casa.	The king who governed the country invited the **general** to eat shrimp and drink a beer.	The lawyer who dealt with the purchase of the building sent the **deed** to the city hall from the Post Office.
El arquitecto les vendió el libro que escribió el año pasado a unos **colegas** que trabajan en otra universidad.	La mamá le pegó la etiqueta que había completado al **cuaderno** que estaba en la mesa.	The bookseller who opened the new store on the corner invited his **colleagues** to the inauguration.	The boy who lives near my house bought a **notebook** to take notes on a field trip.
La compañía le colocó una puerta que manufacturaron la semana pasada al **garaje** de la casa de mis vecinos.	El piloto le añadió la experiencia que había adquirido durante muchos años al **manejo** del avión supersónico.	The hairdresser who bought a car opened the **garage** of her house with her remote control.	The assistant who is in charge of preparing pizzas learned the proper **handling** of the machine to make dough.

La mujer le pegó la cinta aislante que usó el año pasado al **cable** en el cuadro eléctrico.	La instructora le tiró unas gotas de agua que tenía en una botella a la **chispa** del cigarillo.	The postman who works for the city cut the **cable** that hung from the window because it caused a safety hazard.	The man who had taken a course on survival skills created a **spark** to start the fire.
El lingüista le añadió los comentarios que había pensado la noche anterior al **proyecto** escrito por los estudiantes.	El policía le devolvió la paz que había buscado por tanto tiempo al **barrio** con la captura de los ladrones.	The explorer who was interviewed yesterday said that the **project** required more funds for its completion.	The neighbor who lived on the sixth floor of the building decorated the **neighborhood** for the festivities.
El informático le puso el carrete que compró ayer a la **cámara** antes de la sesión fotográfica.	La mujer le attribuyó la causa de las alergías que tiene a la **primavera** y sus flores.	The lawyer who studied at Harvard ordered the **camera** on the Internet because it was less expensive.	The teenager who was in love with a friend waited for the arrival of the **spring** to express his love for her.
El guía le mostró el camino que conocía a los **turistas** que estaban esperando para hacer senderismo.	La vendedora le aplicó un desinfectante que tenía en su bolso a la **herida** que se hizo moviendo unas cajas.	The spy who turned over the data interrogated the **tourists** in Cancun to obtain more information about the case.	The wrestler who defeated the world champion covered his **wound** with a bandage after the fight.
El niño le dio una manzana que tenía en su bolso a la **jirafa** durante la visita al zoo.	El hombre le añadió unas especias que compró en el mercado al **bistec** antes de cocinarlo.	The firemen who arrived at the scene of the accident helped the **giraffe** in danger at the San	The plumber who had three kids at the university prepared a **steak** with vegetables.

Diego Zoo.

El defensor le reveló la información que encontró en el móvil al **reportero** durante la rueda de prensa.	El hombre le dió una langosta que sacó de una caja a la **pescadora** para que la pesara.	The skier who won the medal in the Olympics saw the **reporter** during his jump.	The driver who had the day off for the break called the **fisherwoman** to go out.
El cocinero le puso el trozo de carne que había rellenado el día anterior al **plato** durante el programa de televisión.	El architecto le pegó unas piezas de marmol que había lijado a la **torre** para poder restaurarla.	The woman who opened the cabinet threw a **plate** at her husband because she was angry at him.	The waiter who ran every morning arrived at the **tower** in less than an hour.
El prisionero le dio el tesoro que tenía en el armario al **pirata** durante la pelea en el barco.	El contable le hizo unas correcciones que consideró necesarias al **folio** que le entregó su socio.	The man who sailed across the ocean in his ship killed the **pirate** with a knife.	The trainer who screamed loudly during the game tore the **report** with his notes.
El agente le aplicó la solución que tenía en su maletín a la **pipa** para encontrar las huellas..	La actriz le trajo un collar con diamantes que compró en la joyería a su **perro** después de su viaje.	The dentist who died last week had bought a **pipe** in Turkey for his collection.	The tenant who loves animals always combed his **dog** with a natural bristle brush.

El agricultor le añadió el fertilizante que compró en la tienda a la **planta** que se estaba muriendo.

Los estudiantes le contaron el cuento que oyeron el otro día a la **profesora** de literatura inglesa.

El hombre le atornilló la placa que llegó ayer a la **estatua** de los soldado caidos.

El señor le regaló la entrada que compró para el juego de futbol a un **cliente** de su oficina.

El indio le dio la comida que había encontrado en el suelo a la **cobra** durante el espectáculo.

El geólogo le añadió el estaño que había fundido en su taller al **hierro** para conseguir acero.

La señora le agregó los cordones que tenía en sus zapatillas al **lavado** de la ropa de su hija.

El camarero le pasó una cuchara que tenía detrás de la barra a la **espuma** de la cerveza.

La repostera le colocó unas flores que había visto en una revista al **postre** que preparó para el evento.

La modista le puso unos adornos que había diseñado a la **cinta** del vestido de novia.

The politician who lives in Washington bought a **plant** for his wife because she likes them very much.

The taxi driver who was parked at the corner of the bakery took the **professor** to her house.

The dancer who was at the exhibit will buy a **statue** in Berlin during her next visit.

The woman who had worked at a company that manufactures paper saw her **client** in the mall.

The actress who was on the balcony killed a **cobra** with a shovel that she grabbed from the shed.

The jockey who won the last race of the Kentucky Derby bought **iron** for his stable.

The student who had many chores completed the **wash** with fabric softener before going back home.

The lady who was doing the dishes removed the **foam** from her hands before answering the door.

The dietitian who celebrated his birthday with his friends prepared a **dessert** for the dinner.

The sister who is very meticulous bought a **ribbon** for the bride's veil.

El maestro le dibujó las líneas que consideraba más importantes al **cubo** durante la lección de matemáticas.	El governador le dedicó un tributo que había organizado con sus amigos a la **niñez** del fundador de la universidad.	The assistant who teaches at the school designed the **cube** for the Physics class.	The firefighter who was in danger inside the building remembered his **childhood** as he saved the newborn child.
El artista le envió los cuadros que terminó en su estudio al **organizador** del evento benéfico.	La secretaria le cambió el cartucho que compró en la tienda a la **impresora** antes de irse a su casa.	The nurse who will be working with the cardiologist pushed the **organizer** out the door.	The agent who rented a car at the airport fixed the **printer** for the company.
El fotógrafo le aplicó el barniz que había mezclado en su estudio al **violín** en el auditorio.	La costurera le quería coser unas florecillas que había comprado a la **bragueta** de la chaqueta de su hija.	The actor who lived in Chile bought a **violin** for her daughter's birthday from a famous store.	The goalkeeper who played soccer for the school sewed the **zipper** of his pants before the game.
El artista le aplicó un color extraño que había mezclado al **círculo** en el lienzo.	Las monjas le incluyeron el trabajo que no habían completado al **castigo** de las chicas.	The boy who is in third grade drew a **circle** with his blue pencil and then he cut it and glued it on his book.	The swimmer who ate cakes and cookies received a **punishment** from her coach before the competition.
El hombre le quitó el seguro que había instalado a la **pistola** el día antes del accidente.	El comentarista le quitó la mancha de vino que notó a la **corbata** antes del programa.	The waitress who smokes two packs of cigarettes a day carries a **pistol** in her handbag.	The business man who dressed very well bought a **tie** in a store next to his house.

El espía le facilitó los planos que encontró en el cajón al **oficial** en Irán.

La mujer le puso el queso que compró en la tienda al **brócoli** que estaba preparando para la cena.

La mujer le añadió la crema dulce que compró del granjero a los **caramelos** que estaba preparando.

El gatito le dió el ratón que atrapó anoche a su **familia** durante la cena.

La diplomática le describió el país que visitó al **presidente** que todavía no había estado ahí.

El hombre le puso los muebles que compró en la tienda ayer en su **despacho** del primer piso.

La organización de fútbol le dio el premio que había creado al **árbitro** por su arduo trabajo.

La mujer le agregó el color que había preparado a su **cabellera** mientras estaba en la ducha.

El papá le dio la merienda que calentó en el microondas a los **niños** antes del partido.

La muchacha le contó el secreto que había guardado a su **amiga** de la escuela.

The hostage who was trapped in the bank called the **official** while the thief was distracted.

The girl who always forgets where she leaves her keys went to the store to buy some **broccoli** for the dish that she was preparing for dinner.

The psychologist who studies memory loves it when her participants bring her **caramels** as a present.

The man who loves animals brought home a kitten for his **family** without first telling his wife.

The diplomat who travels to many countries asked the **president** whether she could become a spy.

The man who is very intelligent but messy cleaned his **workroom** before his friend arrived.

The man who likes to exercise decided to become a **referee** after seeing a soccer match.

The girl who is an Olympic gymnast does not want to cut her **hair** despite the fact that it is too long.

The man who lives down the street has three **boys** who follow him everywhere he goes.

The woman who was hired by the company used to be a **friend** of the CEO who lived in Panama.

La matemático le recomendó el programa que había escrito al mejor **estudiante** de su clase.

La señora le explicó la enfermedad que tenía a la **recepcionista** que le dio una cita.

La esposa le puso la funda que había cosido ayer al **sofá** para esconder las manchas.

La policía le dio la muestra de sangre que encontró en la escena del crímen a la **bióloga** para que la analizara.

La oficina de alquiler de autos le dió un GPS que tenía a los **extranjeros** para que no se fueran a perder.

Los niños le enseñaron la leña que habían cortado al **guardabosques** que estaba en la cabaña.

El hombre le añadió algunos ladrillos que recogió ayer al **muro** de la casa que estaba construyendo.

El papá le puso las especias que había mezclado previamente al **cabrito** antes de cocinarlo.

The woman who attended summer camp as a child became the best **student** in her class.

The governor's sister who helped him during his campaign picked up the letters from the **receptionist** before leaving.

The baseball that was signed by a famous player landed on the **sofa** after it fell off the cabinet.

The girl who I met at the store is a **biologist** who makes pharmaceuticals.

The country that was formed ten years ago prohibited **foreigners** from becoming residents.

The police officer who visited the park arrested the **rangers** who were suspected of robbing a bank.

The juror who had been listening to the case for three days straight faced the **wall** when he declared that he needed a rest.

My son who eats meat said that his friend bought **lamb** for the picnic.

El decorador que escribe para la revista predijo que los **problemas** con la pintura serían difícil de resolver.	El cura que da la misa los domingos salvó el **alma** de todos los pecadores la semana pasada.	The business man added several issues that he was worried about to the list of **problems** that will be discussed tomorrow.	According to the legend the devil snatched the happiness that he had longed for from the **soul** that was rising to heaven.
La cajera que trabaja en el banco quemó la **computadora** de su escritorio con su cigarrillo.	El pintor que trabajaba para mi padre por las mañanas sacó la **escalerilla** de la camioneta.	My mother installed some software that she considers very useful on the **computer** in the office.	The technician put the screw that he found yesterday on the **ladder** of the airplane.
El escritor que estaba tratando de buscar ideas para su novela escuchó al **detective** durante el interrogatorio.	El escritor que llegó ayer a Madrid vio la **cabalgata** desde el balcón con sus amigos.	The doctor gave the pictures that he found on the floor to the **detective** at the police station.	The host of the festivities donated some antique chariots that he owned to the **parade** in the small town.
Los profesores que fueron al evento animaron al **atleta** durante su carrera final.	La guía que visitó varias ciudades en Australia preparó un **avestruz** en la cocina.	The nurse gave the medicine that she dissolved in water to the **athlete** with a headache.	The farmer took the egg that he saw in the nest from the **ostrich** before leaving the farm.
La actriz que estudió en Madrid cenó con el **compositor** de San Francisco.	La abuela que había ganado la lotería compró un **congelador** para su casa nueva.	My sister threw a tomato that she got from the refrigerator at the **composer** at the end of the concert.	The girl attached the drawing that she had done at school to the **freezer** in the basement.

El telegrafista que trabaja en la base aérea avisó al **coronel** del ataque que estaban planeando los enemigos.	La enfermera que tenía una hora libre después de su almuerzo limpió la **báscula** en el laboratorio.	The soldier described the beating that he saw to the **colonel** of his division.	The architect added the new base that he designed to the **scale** before showing it to his boss.
El especialista que es muy famoso habló con el **paciente** sobre varios tratamientos para curar su enfermedad.	El astrónomo que trabajaba en el observatorio en Houston tenía un **agujero** en la chaqueta.	The therapist recommended the diet that he had followed himself to the **patient** during the appointment.	The plumber hammered a plank that he had in his van onto the **hole** under the sink.
El jardinero que fue contratado por la escuela preparó una **hamburguesa** para comer con sus amigos.	El médico que bebió demasiadas cervezas en el bar hirió a su **hermanastro** en una pelea.	The mother put the lettuce that she bought at the store on the **hamburger** before serving it.	The man threw a chair that he grabbed from the dining room at his **stepbrother** and then ran away.
El historiador que estaba sentado en el parque sabía todas las **capitales** de los países africanos.	La monja que había perdido la cabeza atacó al **mendigo** en la cocina durante Acción de Gracias.	My son put some pins that he bought yesterday on the **capitals** shown on the map hanging in the classroom.	The priest gave a slice of bread that he had in his backpack to the **beggar** in the street.
La señora que vive en nuestro edificio dejó la **sopa** en la ventana para que se enfriara.	El piloto que dio una celebración en su casa compró el mejor **lomo** y jamón que vendían en la	The girl added the cheese that she liked the most to the **soup** that her mother prepared.	The swimmer removed the fat that he didn't want to cook from the **loin** that he bought at

	tienda.		the butcher's shop.
Los chicos que estaban en el supermercado llamaron al **vendedor** por la ventana.	El estudiante que participó en un experimento devolvió la **encuesta** para el estudio en un sobre.	The farmers gave the apples that they harvested last week to the **vendor** at the market.	The teacher erased the questions that she hadn't written from the **survey** for her students.
El congresista que vive en Minnesota argumentó que su **decisión** fue la correcta dadas las circunstancias.	El escritor que estaba concentrado en su nuevo libro no oyó la **fiesta** en el parque.	Simon added a change that he thought of a few minutes ago to the **decision** made by the committee.	The school board added some restrictions that they felt were necessary to the **party** for the end of the year.
El señor que organizó el evento encontró una **rata** en la despensa de la cocina la semana pasada.	La actriz que amaba su trabajo más que nada invitó al **viajero** a una copa de vino.	The girl gave a piece of bread that she had in the pantry to the **rat** in the apartment.	The gypsy sold a sprig of rosemary that he cut from the tree to the **traveler** that was walking around the square.
El soldado que había luchado en la batalla cosió el **suéter** en su tienda de campaña.	La maestra que quería decorar la habitación de su nueva casa compró un **biombo** el viernes.	My mother sewed some silk flowers that she had designed to the **sweater** that she was knitting for my sister.	The technician installed the new rods that he received yesterday on the **screen** that separates his dining room from his living room.

El contable que tiene tres hijos llamó al **ingeniero** el día de su boda para felicitarlo.	El zoólogo que hacía experimentos con monos frenó su **crecimiento** con el compuesto que había preparado.	The worker sent the advertisements that he selected from the catalog to the **engineer** in Madrid.	During the meeting the doctors put limits that they stipulated as necessary on the **growth** of the hospital.
La panadera que trabaja cerca de la iglesia tiene un **bebé** de cuatro años que ya puede tocar el piano.	El historiador que estaba grabando un programa de TV investigó la **cueva** antes de empezar la grabación.	The uncle gave the crib that he had restored a few months ago to the **baby** for her birthday.	The electrician installed the lights that he bought yesterday on the **cave** behind the Roman ruins.
El arqueólogo que ha dedicado su vida a las ciencias inauguró el **instituto** de Investigación Anatómica.	La cocinera que prerara guisos compró una **papa** en el mercado para hacer la cena.	The geologist sent the stones that he analyzed last month to the **institute** of natural history.	The cook put a spicy sauce that he cooked with his friend on the **potato** that he was going to bake.
El pacifista que había organizado la manifestación pintó el **tanque** con símbolos de paz.	El tartero que tenía una pastelería en la ciudad tiró la **harina** por la calle principal para protestar.	The specialist welded the replacement part that he bought at the store to the **tank** at the campsite.	The cook added the amount of water that he read off the recipe to the **flour** to prepare the dough.
El empresario que había invertido mucho dinero despidió al **director** de la compañía el mes pasado.	La niña que quiere ser artista recibió un premio por su **actuación** en el acto escolar.	The nuns took the quilts that they no longer used to the **director** of the orphanage.	The judges awarded a special prize that they give every year to the best **performance** in the street.

El entrenador que trabaja en la escuela cree que su **estrategia** dará buenos resultados.	El ministro que volvió de sus vacaciones en Italia despidió al **encargado** por haber sido incompetente.	Sandra added some new ideas that she read about in a book to her **strategy** for increasing her success.	The painter gave a picture that he had painted during his free time to the **manager** who assists him.
El ladrón que robó el banco bebió un **café** antes del atraco y luego le dijo a sus amigos lo que hizo.	El público que asistió al desfile de moda reconoció la **belleza** de la modelo de Rumanía.	The guest added filtered water that he got from the refrigerator to the **coffee** that was sitting on the table.	The Romans used to dedicate a tribute that they organized every year to the **beauty** of women.
El cantero que era primo del rey construyó una **catedral** con material reciclado.	El agente que odiaba la actividad física llamó el **ascensor** para ir al primer piso.	The young girls returned the sculptures that they found last week to the **cathedral** during the festivities.	The man put the flyer that he had printed on the **elevator** before leaving the building.
La cuidadora que empezó a trabajar ayer limpió el **teléfono** con agua y jabón para desinfectarlo.	El chico que quiere ser veterinario quería comprar un **caballo** con el dinero de su cuenta de ahorros.	The inspector connected the microphone that he bought at the store to the **telephone** in his house.	The man put the saddle that he designed on the **horse** to make sure it fit.
El mayordomo que trabaja en la mansión llamó al **carpintero** para reparar la puerta principal de la casa de visitas.	La limpiadora que necesitaba ropa para su trabajo pidió unos **calcetines** por internet.	Susana showed the house that she wants to renovate to the **carpenter** who lives next door.	The soldier sewed the patches that he got at the mall to the **socks** in his drawer.

El narrador de noticias que había sido ascendido recientemente animó al **presentador** a actuar con naturalidad en el aire.	La mujer que necesitaba un libro detuvo al **bibliotecario** para hacerle una pregunta.	The teacher gave the discussion topic that he had chosen for the conference to the **presenter** a month ago.	My children asked the questions that they had been thinking of to the **librarian** after she was done talking.
El granjero que trabajaba intensamente en el campo odiaba al **artista** por la vida tan tranquila que llevaba.	El perro que era muy dulce cuidaba a los **ciegos** para mantenerlos fuera de peligro.	The rich businessman gave the photograph that he had taken of his wife to the **artist** in order to paint her portrait.	The millionaire donated the money that he earned over his lifetime to the **blind** to help them.
El dálmata que tenía las orejas caídas se comió el **cereal** que el niño derramó al piso.	La pequeña niña que llevaba su vestido favorito comió **duraznos** en el auto de camino a casa.	The girl added the sugar that she found in the cupboard to her **cereal** after she drank her orange juice.	My mother added the custard that she had prepared to the **peaches** before putting them in the oven.
El soldado que estaba ansioso por demostrar sus capacidades encendió la **dinamita** demasiado pronto y casi pone la misión en peligro.	El hombre que prepara mermeladas hizo un puré de **ciruelas** y lo puso a cocinar.	The thief added the gun powder that he bought the other day to the **dynamite** to create a huge explosion.	The photographer added some wax that he had bought to the **plums** to make them look more appetizing.

La mujer que había hecho la llamada a la policía habló con las **autoridades** acerca del robo cuando llegaron.	La mujer que mantiene su casa muy limpia persuadió los **huéspedes** a que se quitaran los zapatos antes de entrar.	The suspect gave the knife that he had been hiding to the **authorities** when he decided to turn himself in.	The hotel concierge offered the soccer tickets that he had just received to the **guests** after he spilled a vase of flowers all over them.
Las personas que fundaron las Naciones Unidas eran **miembros** de una generación esperanzada de individuos.	El hombre de negocios que trabajaba arduamente vió el **edificio** mientras lo estaban demoliendo.	The magician told the secrets that he had gathered over the years to the **members** of the audience after his show.	The architect added the final details that he had dreamed of to the **building** a month before it opened.
El león que estaba sentado en el campo fijó la mirada en el **antílope** mientras comía hierba junto la laguna.	La zanahoria que el granjero recogió ese día golpeó al **cachorro** haciéndolo geñir.	The veterinarian injected the antibiotics that she had in her cabinet to the **antelope** after it began to shake uncontrollably.	The girl gave the toy that she had bought to her **puppy** after he sat and patiently waited for it.
Mi papá quien ha viajado por el mundo no había visto un **canguro** hasta su viaje a Australia.	La historia que el niño estaba leyendo involucraba un grupo de **duendes** que estaban tratando de salvar el mundo de los gnomos malos.	The farmer put the ointment that she brought with her on the **kangaroo** to make it feel better.	The ogre showed the ring that he was protecting to the **elves** who were hiding him.
